# Clinical significance of EGFR, Her-2 and EGF in oral squamous cell carcinoma: a case control study

**DOI:** 10.1186/1756-9966-29-40

**Published:** 2010-04-29

**Authors:** Vanessa F Bernardes, Frederico O Gleber-Netto, Sílvia F Sousa, Tarcília A Silva, Maria Cássia F Aguiar

**Affiliations:** 1 Department of Oral Surgery and Pathology, School of Dentistry, Universidade Federal de Minas Gerais, Belo Horizonte, MG, Brazil

## Abstract

**Background:**

The erbB receptors and their ligands are involved in the pathogenesis and progression of oral squamous cell carcinoma (OSCC). Although EGFR and Her-2 are frequently overexpressed in OSCC, few studies evaluated these proteins in saliva and their association with the tumor, which may represent potential usefulness in a clinical setting.

**Methods:**

The levels of EGFR, Her-2, and EGF were evaluated in saliva of 46 patients with OSCC before and after the surgical removal of the lesion, as well as in matched healthy controls. The relationship of salivary levels and EGFR and Her-2 immunoexpression in tumor samples with clinicopathological features was analyzed.

**Results:**

EGFR and Her-2 salivary levels did not show difference between to pre-surgery and control groups, however, both demonstrated an increase after surgical removal of the tumor. No association was detectable among receptor salivary levels, tissue expression and clinicopathological features. EGF levels in pre-surgery group were significantly lower when compared to the control group.

**Conclusions:**

EGFR and Her-2 were not considered to be valuable salivary tumor markers in OSCC, however, lower levels of EGF in saliva may suggest a higher susceptibility for OSCC development.

## Background

The epidermal growth factor family of transmembrane tyrosine kinase receptors (erbB receptors) includes four receptors: the epidermal growth factor receptor (EGFR, c-erbB-1, Her-1), c-erbB-2 (Her-2), c-erbB-3 (Her-3), and c-erbB-4 (Her-4) [1,2]. Ligand binding to the erbB receptors leads to the transcription of genes responsible for the inhibition of apoptosis, cell growth, angiogenesis, cell adhesion, cell motility, and invasion, and enhances the malignant potential of epithelial tissues, which in turn overexpress erbB receptors [1,2].

It has been reported that OSCCs present an increase of 42% to 58% in EGFR [3] and 3% to 41% in Her-2 expression [4]. Immunohistochemical staining has been the most common method used to detect overexpression of erbB receptors, however, since its extracelular receptor domain (ECD) can be proteolytically released from the cell surface, this raises the possibility of using serum ECD antigens as diagnostic marker in patient with EGFR and Her-2 overexpressing tumors [5]. However, thenumber of publications that analyzed the levels of erbB receptors in human serum, plasma, or saliva samples is rather small, and the comparison of the published data reveals a great disparity [5,6].

Some studies point toward the need for the simultaneous inclusion of EGF (epidermal growth factor) assessment when analyzing EGF receptors [7]. EGF modulates the growth and differentiation of various cancer cells, as well as normal epithelial cells, and is excreted through human saliva [7,8]. In fact, EGF has been shown to enhance the cell growth of bladder, lung, breast, and colon cancer [8,9].

This study aimed to explore the expression of EGFR, Her-2, and EGF in OSCC. The levels of these proteins in the saliva of patients with OSCC were determined at the moment of diagnosis and six weeks after the surgical removal of the lesion and then compared to healthy matched donors. The immunoexpression of EGFR and Her-2 in tumor samples was evaluated and correlated with the salivary levels of these proteins and the clinicopathological features of the tumors.

## Methods

The protocol of this study was approved by the Research Ethics Committee from Universidade Federal de Minas Gerais, and a signed informed consent was obtained from all the participants.

### Subjects

Patients with a histopathological diagnosis of OSCC were enrolled in the research. Clinical data, such as age, gender, symptoms, location of the tumor, TNM, and tobacco and alcohol habits were obtained from medical records. The saliva was collected at the moment of diagnosis and six weeks after the surgical removal of the tumor.

The control group included healthy individuals without oral lesions and who had been matched by age, sex, and tobacco usage [10].

Patients and controls who showed signs of significant morbidity or active medical problems, such as congestive heart failure, active infection, autoimmune disease, hepatitis, HIV, or abnormal renal function, were excluded from the study.

### Saliva and tumor samples

Subjects were refrained from eating, drinking, smoking, or oral hygiene procedures for at least 1 hour prior to saliva collection. The whole saliva sample was collected for a 5-minute period using a cotton wool swab inserted in the mouth (Salivette^®^, Sarstedt AG & Co., Nümbrecht, Oberbergischer Kreis, Germany). The saliva sample was subsequently diluted (1:1) in a PBS solution containing protease inhibitors (0.1 mM PMSF, 0.1 mM benzethonium chloride, 10 mM EDTA, and 0.01 mg/mL aprotinin A) and 0.05% Tween-20 and was stored at -20°C until analysis. Sections of formalin-fixed, paraffin-embedded incisional biopsy specimens of the tumor were evaluated by H&E staining and used for immunohistochemistry. The histological grade of malignancy was performed employing two parameters of a recognized grading system: degree of keratinization and nuclear pleomorphism [11].

### ELISA

Salivary protein levels were measured by sandwich ELISA, in accordance with the procedures recommended by the manufacturers. The following kits were used: Epidermal Growth Factor Receptor (CBA 018) and c-erbB2/c-neu Rapid Format ELISA kit (QIA10), both from Calbiochem^® ^(Darmstadt, Hessen, Germany) and Human EGF (DuoSet, R&D Systems, Minneapolis, MN, USA).

The total protein content in the saliva was determined using the Bradford method [12] (Sigma, Saint Louis, MO, USA) according to the BSA standard (Fermentas Life Sciences, Vilnius, Lithuania). The total protein content was used to normalize the EGF, EGFR, and Her-2 values for each sample.

### Immunohistochemistry (IHC)

IHC reactions for the detection of EGFR and Her-2 antigens were performed using the monoclonal antibodies clone 31G7 (Zymed Laboratories Inc., San Francisco, CA, USA) and clone CB11 (Novocastra Laboratories, Newcastle upon Tyne, UK), respectively. Sections of oral mucosa and breast carcinoma were used as EGFR and Her-2 positive controls, respectively.

### Evaluation of IHC

EGFR expression was evaluated on the basis of extent and intensity of immunolabeling in tumor cell membranes, classified on a four-point scale: 0 (no labeling, or labeling in < 10% of tumor cells); 1 (weak labeling, homogeneous or patchy, in > 10% of the tumor cells); 2 (moderate labeling, homogeneous or patchy, in > 10% of the tumor cells); 3 (intense labeling, homogeneous or patchy, in > 10% of the tumor cells). These scores were subsequently grouped into two categories: negative (0 or 1) and positive labeling (2 or 3) [13].

The Her-2 protein immunoexpression was analyzed using the American Society of Clinical Oncology/College of American Pathologists (ASCO/CAP) guidelines for Her-2 testing in breast cancer (0, no staining or membrane staining is observed in < 10% of the tumor cells; 1+, faint/barely perceivable membrane staining is detected in > 10% of the tumor cells, and only part of the membrane is stained; 2+, weak to moderate complete membrane staining is observed in > 10% of the tumor cells; 3+, strong complete membrane staining is observed in > 30% of the tumor cells). Data were categorized as negative or positive expression [14].

### Statistical analysis

SPSS (Statistic Package for Social Sciences) 12.0 for Windows (SPSS Inc., Chicago, IL, USA) and Graph Pad Prism 4 were used to analyze the data. Results were expressed as mean ± standard deviation. The Mann Whitney test was used to compare the salivary levels between cases and controls, while the Wilcoxon test was used to compare levels in OSCC patients before and after surgery. The categorical variables were analyzed using the chi-square test. The differences between the values of the groups were considered significant at p < 0.05.

## Results

Patient information and clinicopathological and immunohistochemical data are shown in table 1. The sample consisted of 46 patients with OSCC in varied locations and 46 healthy matched volunteers as the control group (32 male, 14 female). The T-staging and N-staging of the tumors were described according to AJCC (American Joint Committee on Cancer)/UICC (International Union Against Cancer) classification for oral cavity carcinomas [15].

**Table 1:** Clinicopathological features of patients with oral squamous cell carcinoma

Case	Age (years)/Gender*	Site	Tumor differentiation	T status	Nodal metastasis	EGFR expression	Her-2 expression	Follow-up^a^
1	60/M	Tongue	Well	T3-T4	Positive	Positive	Negative	NED
2	60/M	Floor of mouth	Moderately	T3-T4	Positive	Negative	Negative	DOD
3	47/M	Tongue	Poorly	T3-T4	Positive	Positive	Negative	NED
4	55/M	Tongue	Well	T3-T4	Positive	Positive	Negative	DOD
5	43/M	Tongue	Poorly	T3-T4	Positive	Positive	Negative	DOD
6	36/M	Tongue	Well	T3-T4	Positive	Positive	Negative	DOD
7	55/M	Tongue	Moderately	T3-T4	Positive	Negative	Negative	NED
8	44/F	Floor of mouth	Poorly	T1-T2	Positive	Positive	Negative	NED
9	63/M	Tongue	Poorly	T1-T2	Positive	Positive	Negative	NED
10	50/M	Tongue	Poorly	T3-T4	Positive	Negative	Negative	NED
11	55/M	Floor of mouth	Moderately	T1-T2	Negative	Positive	Negative	NED
12	74/M	Floor of mouth	Poorly	T3-T4	Positive	Negative	Negative	DOD
13	58/M	Floor of mouth	Moderately	T3-T4	Negative	Negative	Negative	DOD
14	40/F	Floor of mouth	Poorly	T1-T2	Negative	Negative	Negative	NED
15	57/F	Tongue	Moderately	T1-T2	NA	Negative	Negative	NED
16	56/F	Floor of mouth	Poorly	T3-T4	Positive	Negative	Negative	NED
17	44/M	Floor of mouth	Well	T3-T4	Negative	Negative	Negative	NED
18	62/M	Floor of mouth	Well	T1-T2	Negative	Positive	Negative	NED
19	50/M	Floor of mouth	Moderately	T3-T4	Positive	Negative	Negative	NED
20	54/M	Tongue	Well	T3-T4	Positive	Positive	Negative	NED
21	79/F	Tongue	Well	T1-T2	Negative	Positive	Negative	NED
22	48/M	Tongue	Moderately	T1-T2	Negative	Negative	Negative	NED
23	16/F	Tongue	Moderately	T1-T2	Positive	Negative	Negative	NED
24	49/M	Floor of mouth	Poorly	T3-T4	Positive	Negative	Negative	NED
25	80/M	Floor of mouth	Poorly	T1-T2	Negative	Negative	Negative	NED
26	62/M	Floor of mouth	Poorly	T1-T2	Negative	Negative	Negative	NED
27	72/F	Tongue	Well	T1-T2	Positive	Positive	Negative	NED
28	78/F	Tongue	Well	T1-T2	Negative	Positive	Negative	NED
29	72/F	Tongue	Poorly	T1-T2	Positive	Negative	Negative	DOD
30	48/M	Tongue	Poorly	T3-T4	Positive	Negative	Negative	NED
31	52/F	Tongue	Poorly	T1-T2	Positive	Negative	Negative	NED
32	46/M	Tongue	Poorly	T3-T4	Positive	Negative	Positive	NED
33	61/M	Tongue	Poorly	T3-T4	Positive	Negative	Negative	NED
34	69/M	Floor of mouth	Poorly	T3-T4	Positive	Positive	Negative	NED
35	53/M	Tongue	Well	T1-T2	Positive	Positive	Negative	NED
36	52/M	Tongue	Well	T1-T2	Positive	Positive	Negative	NED
37	35/F	Palate	Well	T1-T2	Negative	Positive	Negative	NED
38	80/F	Gingiva	Moderately	T1-T2	Negative	Negative	Negative	DOD
39	62/F	Gingiva	Well	T1-T2	Negative	Negative	Negative	DOD
40	44/M	Palate	Poorly	T3-T4	Positive	Positive	Negative	NED
41	53/F	Labial mucosa	Well	T1-T2	Positive	Positive	Negative	NED
42	63/M	Gingiva	Moderately	T3-T4	Negative	Positive	Negative	NED
43	45/M	Palate	Moderately	T3-T4	Positive	Positive	Negative	DOD
44	48/M	Palate	Moderately	T3-T4	Positive	Positive	Negative	NED
45	64/M	Palate	Moderately	T3-T4	Positive	Positive	Negative	NED
46	48/M	Gingiva	Moderately	T3-T4	Positive	Negative	Negative	DOD

^a^M: male, F: female, NA: not available; NED: no evidence of disease, DOD: dead of disease

The mean of ages were 55.2 years (range from 16 to 80) and 54.8 years (range from 16 to 86) for the case and control groups, respectively. The tongue and/or floor of the mouth were the most common tumor sites, representing 78.3% of the cases. Eleven (23.9%) patients died from the disease or due to complications during the treatment before completing the period of six weeks after surgery, thus the post-surgery group consisted of 22 individuals. The treatment consisted of surgery followed by postoperative radiotherapy in 16 cases (72.7%), only surgery in 5 cases (22.7%), and surgery together with postoperative radiotherapy plus chemotherapy in one patient (4.6%).

**Figure 1:**
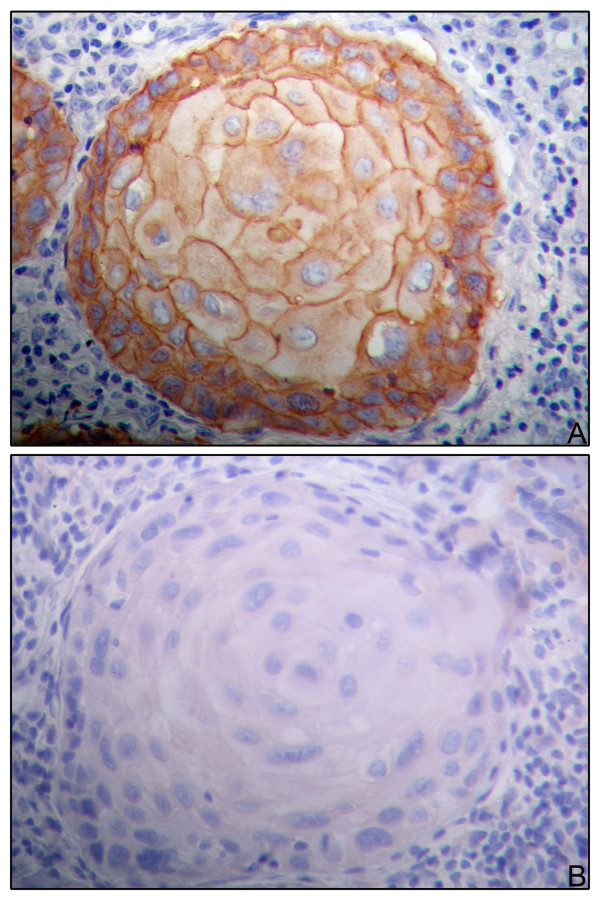
**Immunoexpression of EGFR and Her-2**. **a: **positive immunoexpression of EGFR in OSCC (400×); **b: **negative expression of Her-2 in OSCC (400×).

Fourteen tumors were graded as well differentiated (30.4%), 14 moderately differentiated (30.4%), and 18 poorly differentiated (39.2%). The EGFR immunohistochemical expression was considered positive in 23 (50%) cases (Figure 1a), while 23 tumors (50%) proved to be negative (Table 1). The well differentiated tumors were more frequently positive for EGFR than were the others (p < 0.05) The Her-2 protein expression was found to be negative in 45 (97.8%) cases (Table 1) (Figure 1b).

**Figure 2:**
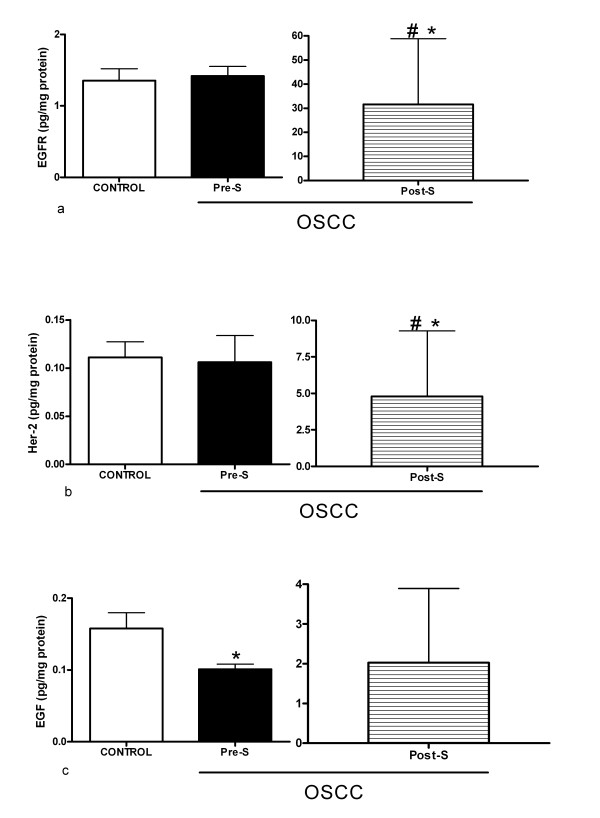
**Salivary levels of EGFR, Her-2 and EGF**. **a: **Salivary levels with standard deviation of EGFR in the control and OSCC groups; **b: **salivary levels with standard deviation of Her -2 in the control and OSCC groups; **c: **salivary levels with standard deviation of EGF in the control and OSCC groups. OSCC: oral squamous cell carcinoma; Pre-S: pre-surgery; Post-S: post-surgery; *:OSCC vs. control group (p < 0.05); ^#^: pre-surgery vs. post-surgery (p < 0.05).

Salivary levels of EGFR, Her-2, and EGF are represented in figure 2a, b, c. EGFR and Her-2 salivary levels did not show difference between to pre-surgery and control groups. The measures 6 weeks after surgery showed a significant increase of EGFR and Her-2 (p < 0.05). The salivary levels of EGF in the pre-surgery group, as compared to the control group, were significantly lower. A tendency toward an increase in EGF levels after surgery as regards the pre-surgery and control groups could be observed, but the difference was not statistically significant (p > 0.05). The EGF/EGFR ratio in the pre-surgery group (0.09 ± 0.05) was significantly lower than that in the control group (0.12 ± 0.05). The post-surgery group presented a significantly higher ratio (2.88 ± 15.74) in relation to the pre-surgery group (p < 0.05) and showed a trend towards a higher ratio when compared to the control (p = 0.057). The EGF/Her-2 ratio presented significant differences when comparing the post-surgery group (29.49 ± 193.67) to the control group (1.91 ± 1.48) and the post-surgery group to the pre-surgery group (1.74 ± 1.27) (p < 0.05).

There was no significant association between EGFR, Her-2, and EGF salivary levels and the immunoexpression of the proteins EGFR and Her-2 in tumor specimens (p > 0.05). The salivary levels of the proteins were not associated with clinicopathological features, such as patient age, smoking habit, site, histological grading, T status, or nodal involvement of the tumor (p > 0.05).

## Discussion

An increased attention has been focused on the role of growth factors and their receptors in pathogenesis of HNSCC (head and neck squamous cell carcinoma) and as potencial targets for new therapies [16-18]. In the present study, EGFR overexpression was found in 50% of OSCC, while 97.8% of the tumor specimens were negative for Her-2. Although EGFR overexpression has been reported to be a hallmark of OSCC [5,19,20], investigations on Her-2 in OSCC have described protein overexpression in a very few tumour specimens, which did not appear to be of prognostic relevance [5,17,21,22].

Some studies have reported an association between the overexpression of EGFR and poor tumor differentiation in OSCC [20]. Conversely, our results demonstrated an increase of EGFR expression in well differentiated tumors, as has been reported in prior literature [23]. A possible explanation is that this receptor may be related to the degree of differentiation of neoplastic keratinocytes [23].

In the present study, salivary EGFR and Her-2 levels were not elevated in patients with OSCC. Moreover, no significant association was found between the salivary levels of the proteins and clinicopathological data, such as patient age, smoking habit, site, histological grading, T status, or nodal involvement of the tumor and most notably, no diferences in salivary levels could be observed in patients with immunohistochemically positive nor negative tumors. In a similar study evaluating breast cancer [24], the authors found no association between the levels of Her-2 in the serum and those in the tumor. While the activation of EGFR and Her-2 on the cell surface of the head and neck tumors has proven to lead to tumor growth, these are not necessarily expressed in altered levels, nor released into the saliva of OSCC patients. It is also important to consider that epithelial tumours present different capacities to shed EGFR and Her-2 ECD from the cell membrane to saliva or to metabolize these proteins [25]. In addition, certain factors not related to the cancer may influence the Her-2 ECD levels, such as hormones, nonmalignant hepatic disorders and others [6,26,27]. Finally, some studies have suggested that protein levels in the serum, as compared to those in the tissue, tend to be lower. The authors associated the results with the methods used to determine cut-off points in the serum, as compared to those in the tissue (usually through immunohistochemical staining using visual analysis) [28].

EGFR and Her-2 showed elevated levels after surgical removal. The increased ratio of EGF/EGFR and EGF/Her-2 in post-surgery patients may reflect the role of EGF and metaloproteinases in healing [29]. In addition, the metaloproteinases (MMPs), responsible for the degradation of the extracellular matrix and remodeling, are also involved in the release of ECD, whereas the increased levels of EGFR, Her-2, and EGF after the removal of the tumor may be indicative of up-regulated MMP activity during healing [30].

The salivary levels of EGF in the pre-surgery group, as compared to the control group, were significantly lower. EGF is the major ligand for EGFR and a mitogenic factor which stimulates the cell division of various tissues and plays an important role in maintaining the anatomic continuity of the oral cavity's mucous membrane [7]. The low concentration of EGF in cancer patients observed in this study is in agreement with previous data concerning the serum of thyroid carcinoma [31]. Our results from pre-surgery patients suggest that the impaired ability to heal oral mucosa damage in neoplastic diseases may be related to the low EGF concentration in the saliva [32-34]. Another hypothesis to explain the lower concentration of EGF in the saliva of patients with OSCC may be the correlation between the EGF and ligands competing for EGFR [7]. Therefore, it is suggested that the lower EGF/EGFR ratio in OSCC patients, as compared to the controls, observed in this study may represent a higher receptor-ligand affinity due to the tumoral process [33]. Expression of a high number of receptors or truncated receptors on the surface of tumor cells can increase the sensitivity to low concentrations of host- or tumor-derived growth factors [32].

## Conclusions

These findings suggest that the use of EGFR and Her-2 as salivary markers of OSCC is not recommended because no significant preoperative elevation and no association to clinicopathological features were found. The lower EGF concentration in the saliva of pre-surgery patients and its growing tendency after surgery may suggest an important role for this factor in oral cavity carcinoma development as well as in the healing of oral mucosa. Further studies are needed due to the complexity of the system at the receptor and ligand levels and the integrated biological functions of the erbB family in oral squamous cell carcinomas.

## Competing interests

The authors declare that they have no competing interests.

## Authors' contributions

VFB carried out the literature research, data acquisition, experimental work and the preparation of the manuscript. FOGN and SFS participated of the sample collection and of the experiments. TAS contributed to statistical and data analysis, besides manuscript editing and review. MCFA carried out the conception and the design of the study, manuscript editing and review and is the guarantor of the integrity of the research. All authors read and approved the final manuscript.
